# STEP activation by Gαq coupled GPCRs opposes Src regulation of NMDA receptors containing the GluN2A subunit

**DOI:** 10.1038/srep36684

**Published:** 2016-11-18

**Authors:** Meng Tian, Jian Xu, Gang Lei, Paul J. Lombroso, Michael F. Jackson, John F. MacDonald

**Affiliations:** 1 Molecular Medicine, Robarts Research Institute, Schulich School of Medicine, the University of Western Ontario, London, Ontario, N6A 5B7, Canada; 2 Child Study Center, Yale University School of Medicine, 230 South Frontage Rd, New Haven, CT, 06520, USA; 3 Departments of Psychiatry, and Neuroscience, Yale University School of Medicine, 230 South Frontage Rd, New Haven, CT, 06520, USA; 4 Department of Pharmacology and Therapeutics, College of Medicine, University of Manitoba, Winnipeg, Manitoba R3E 0T6, Canada; 5 Neuroscience Research Program, Kleysen Institute for Advanced Medicine, University of Manitoba, Winnipeg, Manitoba, R3E 3J7, Canada; 6 Department of Physiology and Pharmacology, Schulich School of Medicine, the University of Western Ontario, London, Ontario, N6A 5C1, Canada.

## Abstract

*N*-methyl-D-aspartate receptors (NMDARs) are necessary for the induction of synaptic plasticity and for the consolidation of learning and memory. NMDAR function is tightly regulated by functionally opposed families of kinases and phosphatases. Herein we show that the striatal-enriched protein tyrosine phosphatase (STEP) is recruited by Gα_q_-coupled receptors, including the M1 muscarinic acetylcholine receptor (M1R), and opposes the Src tyrosine kinase-mediated increase in the function of NMDARs composed of GluN2A. STEP activation by M1R stimulation requires IP_3_Rs and can depress NMDA-evoked currents with modest intracellular Ca^2+^ buffering. Src recruitment by M1R stimulation requires coincident NMDAR activation and can augment NMDA-evoked currents with high intracellular Ca^2+^ buffering. Our findings suggest that Src and STEP recruitment is contingent on differing intracellular Ca^2+^ dynamics that dictate whether NMDAR function is augmented or depressed following M1R stimulation.

Postsynaptic NMDA receptors in CA1 hippocampal neurons are required for the induction of synaptic plasticity and contribute to the consolidation of hippocampal based learning and memory. The predominant subtypes of NMDARs at these synapses are those containing either GluN2A or GluN2B subunits^1^. A wealth of evidence supports the view that each receptor population possesses distinct subcellular distributions, with GluN2A being predominantly synaptic and GluN2B predominantly extrasynaptic^2^,^3^,^4^, albeit with varying degrees of overlap. Importantly, neuronal signalling downstream of GluN2ARs and GluN2BRs mediate distinct functional outcomes, not only within the hippocampus but within other CNS regions as well. This includes opposing contributions to the induction of synaptic plasticity, neuronal survival and cell death^1^,^2^,^5^. Evidence suggests that GluN2ARs and GluN2BRs are differentially regulated via distinct cell signalling cascades that include the Src family kinases (SFKs) members Src and Fyn. For example, signalling through G-protein coupled receptors (GPCRs) of the Gαq category selectively enhances GluN2ARs as a consequence of Src activation whereas signalling through GPCR of the Gαs category selectively enhances GluN2BRs as a consequence of Fyn stimulation^6^.

The cholinergic innervation of the hippocampus is important for controlling various aspects of learning and memory and its loss is associated with conditions such as Alzheimer’s disease^7^. Activation of Gαq-coupled muscarinic acetylcholine receptors (mAchRs) potentiates NMDARs^8^,^9^,^10^ and enhances NMDAR-dependent LTP^11^. Paradoxically, however, they can also depress NMDARs^9^,^12^ and promote the induction of NMDAR-dependent LTD^13^,^14^. More recently, mAChR stimulation has been shown to induce LTD of NMDAR-mediated synaptic transmission by a mechanism involving AP-2 and dynamin-dependent internalization of NMDARs^15^. Overall, these results present a conundrum in that mAChRs both enhance or depress NMDAR currents in hippocampal neurons.

We have resolved this conundrum by testing the hypothesis that Gαq GPCRs, including M1 mAChRs (M1Rs), recruit both Src kinase and the striatal-enriched protein tyrosine phosphatase (STEP) concurrently. The balance between kinase and phosphatase activity ultimately determines the degree of phosphorylation of NMDAR subunits and by extension their level of activity. We now report that a key factor in determining the balance between Src and STEP activation downstream of Gαq GPCR stimulation is the intracellular concentration of Ca^2+^. Low Ca^2+^ (or high Ca^2+^ buffering) favors the activity of Src and leads to a strong potentiation of NMDA-evoked currents by muscarine. This potentiation required Ca^2+^ entry via NMDARs and was effected specifically through a Src-mediated increase of GluN2AR function, without participation of Fyn or GluN2BRs. Conversely, elevated intracellular Ca^2+^ (or low Ca^2+^ buffering) favours STEP activation and leads to a muscarine induced depression of NMDARs. This STEP-mediated depression of NMDA-evoked currents required inositol triphosphate receptor (IP3R) stimulation. Our results demonstrate that intracellular Ca^2+^ dynamics play a key role in controlling Gαq-coupled GPCR-induced changes in NMDAR function through a differential recruitment of Src and STEP. The mechanisms described are likely to be important determinants of metaplasticity.

## Results

### NMDA receptor-mediated currents are constrained by a Gαq-coupled GPCR stimulated tyrosine phosphatase

**Figure 1:**
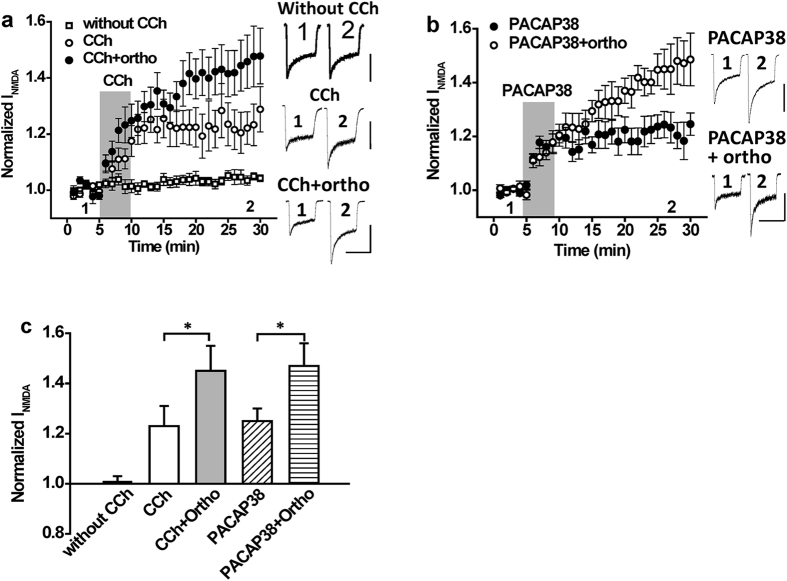
The potentiation of NMDAR currents by Gαq coupled receptor stimulation is increased by inhibiting protein tyrosine phosphatases. (**a**) Application of CCh (5 μM; timing indicated by the shaded region) potentiates NMDAR currents (n = 7, 1.23 ± 0.08). The potentiation by CCh is increased when applied in the presence of the tyrosine phosphatase inhibitor orthovanadate (10 μM; n = 7, 1.45 ± 0.10, *P* < 0.05 compared with CCh alone). In the absence of CCh treatment, NMDA currents remain stable (without CCh; n = 8, 1.01 ± 0.02). (**b**) Application of PACAP38 (1 nM; timing indicated by the shaded region) potentiates NMDAR currents (n = 6, 1.25 ± 0.05). The potentiation by PACAP38 is increased when applied in the presence of the tyrosine phosphatase inhibitor orthovanadate (10 μM; n = 6, 1.47 ± 0.09, *P* < 0.05 compared with PACAP38 alone). (**c)** Summary bar graph of data in (**a,b**). *Indicates P < 0.05. Calibration bars: 3s; (**a**) without CCh 500 pA, CCh 300 pA, CCh + Ortho 400 pA; (**b**) PACAP38 300 pA, PACAP38 + Ortho 600 pA.

We previously showed that muscarine potentiates NMDA-evoked currents in hippocampal CA1 neurons by recruiting Src^8^. In contrast, muscarine is reported to consistently inhibit NMDAR currents in hippocampal CA3 neurons^9^,^12^. When hippocampal CA3 neurons were treated with a non-specific tyrosine phosphatase inhibitor (pervanadate), the inhibition of NMDAR currents by muscarine was converted to a potentiation^12^. These finding suggest that M1Rs can either potentiate or inhibit NMDA-evoked currents through mechanisms involving Src or an unidentified protein tyrosine phosphatase (PTP). We therefore investigated whether PTPs limit the ability of Src kinase to enhance NMDAR currents downstream of Gαq-coupled GPCR stimulation in CA1 hippocampal neurons. Stimulation of mAChRs with the non-selective muscarinic agonist carbachol (CCh, 5 μM) potentiated peak responses to rapid applications of NMDA (50 μM, 0.5 μM glycine; Fig. 1a,c, n = 7, *P* < 0.05 compared with baseline). When applied in the presence of the non-specific PTP inhibitor sodium orthovanadate (10 μM), the potentiation of NMDAR currents by CCh was substantially enhanced (Fig. 1a,c, n = 7, *P* < 0.05 compared with CCh alone). Sodium orthovanadate did not influence peak NMDAR currents when applied alone (Supplementary Fig. 1, n = 4, *P* > 0.05 compared with baseline). This suggests that a PTP is recruited downstream of mAChR stimulation to oppose the potentiation by Src kinase. To determine if PTPs are recruited downstream of another Gαq-coupled GPCR we examined the effects of PTP inhibition on NMDA-evoked currents during applications of the selective PAC_1_R agonist PACAP38 (1 nM). Sodium orthovanadate enhanced the potentiation of NMDARs by PACAP38 implying that PAC_1_Rs also activate an endogenous PTP that limits the extent of NMDA-evoked current potentiation (Fig. 1b,c, n = 6, *P* < 0.05 compared with PACAP38 alone).

### STEP activation by M1 muscarinic receptors limits Src potentiation of GluN2A containing NMDARs

**Figure 2:**
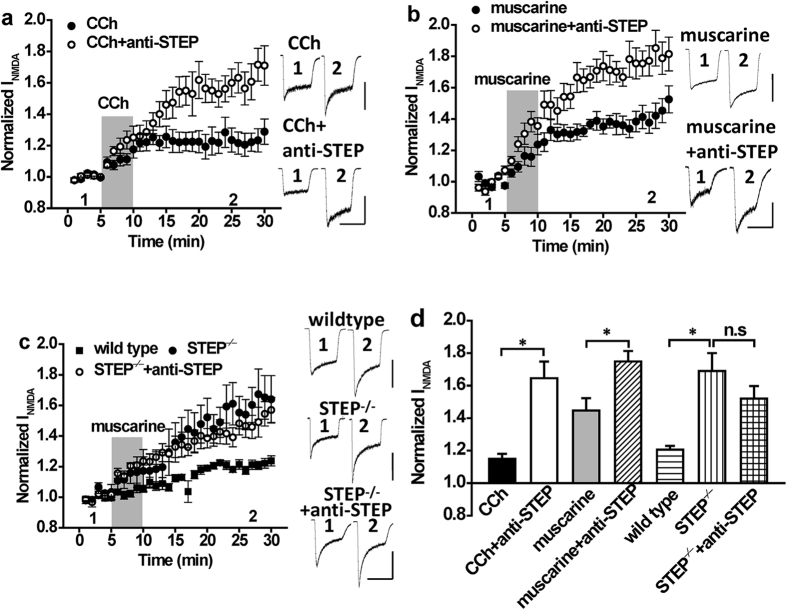
The potentiation of NMDAR currents by mAchR agonists is increased by inhibition or genetic deletion of STEP. (**a**) Application of CCh (5 μM; timing indicated by the shaded region) potentiates NMDA evoked currents (n = 5, 1.15 ± 0.03). The potentiation of NMDAR currents by CCh is increased by anti-STEP (n = 5, 1.65 ± 0.10, *P* < 0.05 compared with CCh). (**b**) Application of muscarine (10 μM; timing indicated by the shaded region) potentiates NMDA evoked currents (n = 5, 1.44 ± 0.08) and this potentiation is increased by anti-STEP (n = 6, 1.71 ± 0.07, *P* < 0.05 compared with muscarine). (**c**) The potentiation of NMDAR currents by muscarine is greater in neurons from STEP KO (n = 6, 1.69 ± 0.13, *P* < 0.05 compared with wild type) than in neurons from wild-type mice (n = 10, 1.21 ± 0.02). Anti-STEP fails to enhance muscarine potentiated NMDAR currents in neurons isolated from STEP KO mice (n = 5, 1.51 ± 0.08, *P* > 0.05 compared with STEP KO). (**d**) Summary bar graph of data in (**a**–**c**). *Indicates P < 0.05, n.s. indicates *P* > 0.05. Calibration bars: 3s; (**a**) CCh 300 pA, CCh + anti-STEP 300 pA; (**b**) Muscarine 500 pA, muscarine + anti-STEP 400 pA; (**c**) wild type 600 pA, STEP^−/−^ 200 pA, STEP^−/−^ + anti-STEP 200 pA.

In considering the identity of the PTP that opposes the enhancement of NMDAR currents in response to Gαq-coupled GPCR stimulation, one candidate is STEP^16^. Tyrosine dephosphorylation of the STEP substrate GluN2B depresses NMDAR function in hippocampal neurons^17^ and promotes internalization of GluN2B-containing NMDARs (GluN2BRs) following exposure to β-amyloid^18^,^19^,^20^. To determine if STEP is involved, we supplemented the patch pipette solution with anti-STEP (1:400 dilution), a functional inhibitory antibody previously shown to selectively inhibit the activity of STEP^17^,^21^. The presence of anti-STEP dramatically enhanced the potentiation of these currents by CCh and by the mAchR-selective agonist muscarine (10 μM; Fig. 2a,b,d, CCh + anti-STEP: n = 5, *P* < 0.05 compared with CCh alone; muscarine + anti-STEP: n = 6, *P* < 0.05 compared with muscarine alone). In contrast, no time-dependent change in the amplitude of NMDAR currents occurred when anti-STEP was applied alone (Supplementary Fig. 1, n = 4, *P* > 0.05 compared with baseline). Confirming STEP involvement, the enhancement of NMDAR currents by muscarine was greater in neurons from STEP^−/−^ mice (Fig. 2c,d, n = 6, *P* < 0.05 compared with wildtype) when compared to those from WT (Fig. 2c,d, n = 6, *P* < 0.05 compared with baseline). Importantly, anti-STEP did not affect the potentiation of NMDAR currents by muscarine in neurons from STEP^−/−^ mice (Fig. 2c,d, STEP^−/−^ + anti-STEP: n = 5, *P* > 0.05 compared with STEP^−/−^ mice), confirming the functional specificity of anti-STEP. The potentiation of NMDAR currents by both CCh and muscarine is mediated via the M1 subtype of mAChRs (M1Rs) as the potentiation was replicated by application of the M1R agonist, xanomeline (10 μM), and could be blocked by the M1R antagonist, pirenzepine (10 μM; Supplementary Fig. 2a,b, xanomeline: n = 5, *P* < 0.05 compared with baseline; xanomeline + anti-STEP: n = 5, *P* < 0.05 compared with xanomeline alone; muscarine + anti-STEP + pirenzepine: n = 3, *P* > 0.05 compared with baseline; CCh + anti-STEP + pirenzepine: n = 4, *P* > 0.05 compared with baseline).

**Figure 3:**
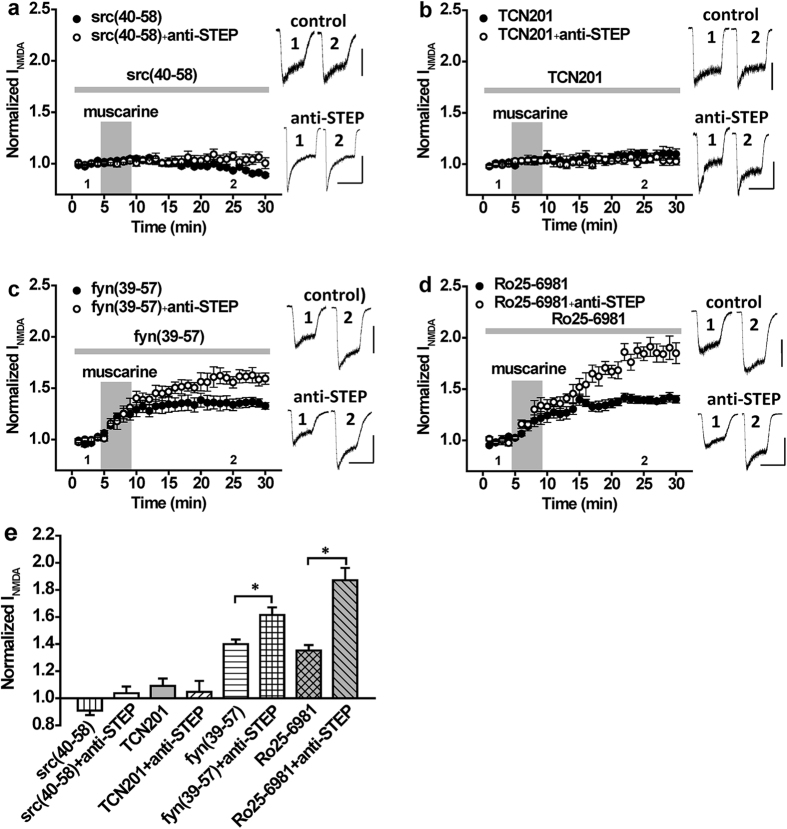
The potentiation of NMDAR currents by mAchR stimulation and its increase by anti-STEP is contingent on GluN2A subunit containing NMDARs and Src kinase. (**a**) Muscarine application (10 μM; timing indicated by the shaded region) fails to potentiate NMDAR currents in the presence of Src(40–58) (n = 6, 0.91 ± 0.02) as well as in the presence of Src(40–58) and anti-STEP (n = 6, 1.03 ± 0.05). (**b**) Muscarine application (10 μM) fails to potentiate NMDAR currents in the presence of the selective GluN2A receptor allosteric inhibitor TCN201 (10 μM; n = 6, 1.09 ± 0.05) as well as in the presence of TCN201 and anti-STEP (n = 5, 1.04 ± 0.08). (**c**) Muscarine application (10 μM) potentiates NMDAR currents in the presence of Fyn(39–57) (n = 5, 1.40 ± 0.03) and this potentiation is increased by anti-STEP (n = 5, 1.61 ± 0.06, *P* < 0.05 compared with muscarine). (**d**) Muscarine application (10 μM) potentiates NMDAR currents in the presence of Ro25-6981 (n = 6, 1.35 ± 0.04) and this potentiation is increased by anti-STEP (n = 5, 1.87 ± 0.10, *P* < 0.05 compared with Ro25-6981). (**e**) Summary bar graph of data in (**a–d**). *Indicates *P* < 0.05. Calibration bars: 3s; (**a**) src(40–58) 100 pA, src(40–58) + anti-STEP 300 pA; (**b**) TCN201 150 pA, TCN201 + anti-STEP 150 pA; (**c**) fyn(39–57)) 400 pA, fyn(39–57) + anti-STEP 400 pA; (**d**) Ro25-6981 200 pA, Ro25-6981 + anti-STEP 200 pA.

We previously demonstrated that PACAP38 enhances NMDAR currents via a Src-dependent phosphorylation of GluN2A subunits^6^ and therefore examined if a similar mechanism was in play for mAChRs. To do so, we used a Src peptide (Src40-58) that disrupts the association between Src and GluN2A thereby preventing its Tyr phosphorylation^22^. This peptide selectively blocks the potentiation of GluN2ARs by Src, but not of GluN2BRs by Fyn^6^. Treatment with Src40-58 (25 ng/ml) blocked both the potentiation of NMDA responses by muscarine as well as the additional enhancement in the presence of anti-STEP (Fig. 3a,e, n = 6, *P* > 0.05 compared with baseline). In contrast, inclusion of Fyn39-57 (25 ng/ml), a Fyn interfering peptide, neither blocked the muscarine-induced potentiation nor the anti-STEP enhancement of these currents (Fig. 3c,e, fyn(39–57): n = 5, *P* < 0.05 compared with baseline; anti-STEP + fyn(39–57): n = 5, *P* < 0.05 compared with fyn(39–57)). This peptide selectively blocks the potentiation of GluN2BRs by Fyn, but not of GluN2ARs by Src^6^.

To demonstrate the involvement of GluN2ARs in mediating the enhancement of NMDAR currents by muscarine we employed TCN201 (10 μM), a highly selective allosteric inhibitor of GluN2ARs^23^,^24^,^25^. When neurons were bathed in a solution containing this agent, muscarine failed to potentiate NMDAR currents, even when anti-STEP was included in the patch pipette (Fig. 3b,e, TCN201: n = 6, *P* > 0.05 compared with baseline; anti-STEP + TCN201: n = 5, *P* > 0.05 compared with baseline). Conversely, block of GluN2BRs by the selective GluN2BR antagonist Ro 25-6981 (0.5 μM)^26^,^27^ had no effect on the muscarinic-induced potentiation of NMDA currents nor on the enhancement of this action by anti-STEP (Fig. 3d,e, Ro25-6981: n = 6, *P* < 0.05 compared with baseline; anti-STEP + Ro25-6981: n = 5, *P* < 0.05 compared with Ro25-6981).

Previously we showed that the dopamine 1 receptor (D1R), a Gαs-dependent GPCR, regulates NMDA responses in CA1 neurons through Fyn kinase and GluN2BRs but not Src and GluN2ARs^6^. Although Fyn and GluN2BRs are well recognized STEP substrates, D1R stimulation has been shown to suppress STEP activity through PKA-mediated phosphorylation. We therefore predicted that augmented GluN2BR function resulting from D1R-mediated Fyn activation would not be subject to the opposing influence of STEP. Confirming this prediction, the presence of anti-STEP had no effect on the potentiation of NMDA responses resulting from D1R stimulation by SKF81297 (Supplementary Fig. 3, control: n = 6, *P* < 0.05 compared with baseline; anti-STEP: n = 6, *P* > 0.05 compared with SKF81297).

### Intracellular Ca^2+^ regulates the direction of change in NMDAR function imposed by STEP or Src

**Figure 4:**
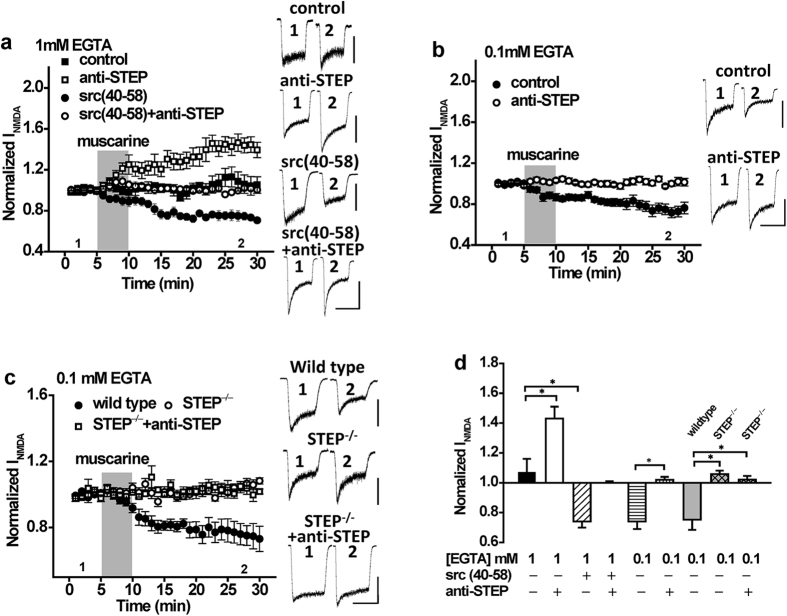
The direction of change in NMDAR function as a consequence of mAchR stimulation is influenced by the intracellular concentration of EGTA. (**a**) With 1 mM intracellular EGTA, muscarine application (10 μM; timing indicated by the shaded region) fails to potentiate NMDAR currents (n = 5, 1.07 ± 0.10), but can do so in the presence of anti-STEP (n = 6, 1.43 ± 0.09, *P* < 0.05 compared with muscarine). Conversely, with 1 mM EGTA, muscarine depresses NMDAR currents in the presence of Src (40–58) (n = 5, 0.74 ± 0.04, *P* < 0.05 compared with muscarine) but not in the presence of both Src(40–58) and anti-STEP (n = 6, 1.00 ± 0.01). (**b**) With 0.1 mM intracellular EGTA, the depression of NMDAR currents by muscarine (n = 5, 0.74 ± 0.05) is inhibited by anti-STEP (n = 7, 1.02 ± 0.02). (**c**) With 0.1 mM intracellular EGTA, muscarine application depresses NMDAR currents in neurons from wild type mice (n = 7, 0.75 ± 0.07) but not in neurons from STEP KO mice (n = 7, 1.05 ± 0.02, *p* > 0.05 compared with baseline). Anti-STEP had no effects on NMDAR currents in STEP KO mice (n = 5, 1.02 ± 0.02, *P* > 0.05 compared with STEP KO mice without anti-STEP). (**d**) Summary bar graph of data in (**a–c**). *Indicates *p* < 0.05. Values represent the average of peak current amplitudes recorded between 20–25 min normalized to the average of peak current amplitudes recorded during the first 5 min of recording, prior to muscarine application. Calibration bars: 3s; (**a**) control 100 pA, anti-STEP 300 pA, Src(40–58) 200 pA, Src(40–58) + anti-STEP 200 pA. (**b**) control 300 pA, anti-STEP 400 pA. (**c**) wild type 600 pA, STEP^−/−^ 200 pA, STEP^−/−^ + anti-STEP 200 pA.

Our results show that M1R stimulation leads to a Src-dependent potentiation of GluN2ARs in CA1 pyramidal neurons in spite of a paradoxical stimulation of STEP. In recordings from isolated CA1 neurons, we employed a relatively high concentration of the slow Ca^2+^ chelator EGTA (11 mM EGTA). In contrast, Jo and colleagues 2010 employed a much lower concentration of EGTA (0.5 mM) in their patch recordings and demonstrated that CCh induced an inhibition of synaptic NMDARs. This suggested the possibility that the balance between effects of Src and STEP on NMDAR function may be influenced by the degree of Ca^2+^ buffering. Therefore, we performed a series of recordings in which we varied the amount of Ca^2+^ buffering in the patch solutions. When EGTA was reduced to 1 mM, applications of muscarine failed to potentiate peak NMDARs (Fig. 4a,d, n = 5, *P* > 0.05 compared with baseline). However, when Src(40–58) was included in the patch pipette to block Src, muscarine induced a robust inhibition of NMDARs (Fig. 4a,d, n = 5, *P* < 0.05 compared with muscarine alone). Conversely, when anti-STEP was administered to the cell interior, muscarine now potentiated NMDAR currents (Fig. 4a,d, n = 6, *P* < 0.05 compared with muscarine alone). Potentiation by anti-STEP was blocked by Src(40–58) (Fig. 4a,d, n = 6, *P* > 0.05 compared with baseline). Given these results, we further reduced the concentration of EGTA to 0.1 mM and under these recording conditions muscarine exclusively inhibited peak NMDARs (Fig. 4b,d, n = 5, *P* < 0.05 compared with baseline). Importantly, this inhibition was blocked by including anti-STEP in the patch pipette (Fig. 4b,d, n = 7, *P* > 0.05 compared with baseline) and was absent in neurons isolated from STEP^−/−^ mice (Fig. 4c,d, n = 7, *P* > 0.05 compared with baseline). These results demonstrate that Ca^2+^ buffer capacity in neurons determines whether the M1R stimulation predominantly activates STEP or Src, leading to inhibition or potentiation, respectively.

**Figure 5:**
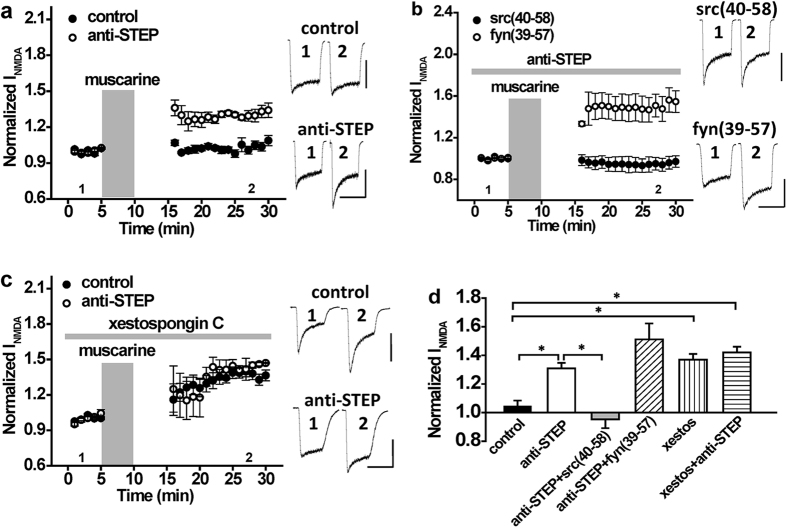
The potentiation of NMDA currents by mAchR stimulation requires NMDAR co-stimulation. (**a**) With 11 mM intracellular EGTA, muscarine application (10 μM; timing indicated by the shaded region) without NMDAR co-stimulation fails to potentiate NMDAR currents (n = 5, 1.02 ± 0.02). Muscarine without NMDAR co-stimulation can potentiate NMDAR currents in the presence of anti-STEP (n = 7, 1.30 ± 0.02, *P* < 0.05 compared with muscarine). (**b**) With 11 mM intracellular EGTA, muscarine without NMDAR co-stimulation can potentiate NMDAR currents in the presence of anti-STEP and Fyn(39–57) (n = 6, 1.51 ± 0.11, *P* < 0.05 compared with baseline), but not in the presence of anti-STEP and Src(40–58) (n = 6, 0.95 ± 0.06, *P* > 0.05 compared with baseline). (**c**) With 11 mM intracellular EGTA and in the absence of NMDAR co-stimulation, muscarine can potentiate NMDAR currents in the presence of xestospongin C (10 μM; n = 5, 1.37 ± 0.05). In the presence of xestospongin C, no further increase of NMDAR current amplitude is observed with anti-STEP (n = 5, 1.41 ± 0.06, *P* > 0.05 compared with anti-STEP). (**d**) Summary bar graph of data in (**a**–**c)**. *Indicates *P* < 0.05. Calibration bars: 3s; (**a**) control 400 pA, anti-STEP 400 pA (**b**) Src(40–58) 500 pA, Fyn(39–57) 500 pA (**c**) control 600 pA, anti-STEP 500 pA.

We next sought to determine the source of the Ca^2+^ transients that regulate Src and STEP signalling, albeit at different intracellular Ca^2+^ concentrations. Two primary sources likely contribute to Ca^2+^ elevations in our recordings; entry via NMDARs and release from intracellular stores by M1R-mediated activation of IP_3_Rs. In previous experiments, muscarine and NMDA were simultaneously applied to isolated neurons. To determine the contribution of Ca^2+^ influx via NMDA receptors in this paradigm, we stopped NMDA exposure during muscarine application and waited another 5 min to thoroughly wash out any residual muscarine before re-commencing applications of NMDA. With these conditions the muscarine potentiation of NMDAR currents was absent (Fig. 5a,d, control: n = 5, *P* > 0.05 compared with baseline). This implies that the potentiation by muscarine is dependent in part upon the entry of Ca^2+^ via NMDARs. When repeated in the presence of anti-STEP, a substantial potentiation of NMDAR currents was now observed (Fig. 5a,d, anti-STEP: n = 7, *P* < 0.05 compared with baseline). As the application of anti-STEP alone does not potentiate NMDAR currents (Supplementary Fig. 1, n = 4, *P* > 0.05 compared with baseline), this suggested to us that modest activation of Src likely occurred when muscarine was applied in the absence of concurrent NMDAR stimulation. This was confirmed by our finding that anti-STEP facilitated potentiation of NMDARs by muscarine (without NMDAR co-stimulation) could be prevented by Src40-58 (Fig. 5b,d, anti-STEP + Src(40–58): n = 6, *P* > 0.05 compared with baseline), but not by Fyn39-57 (Fig. 5b,d, anti-STEP + Fyn(39–57): n = 6, *P* < 0.05 compared with baseline). Given that Ca^2+^ influx via NMDARs is important for robust Src recruitment but not for STEP, we questioned whether IP_3_R-regulated Ca^2+^ stores contributed to STEP recruitment following M1R stimulation. In the presence of xestospongin C (10 μM; an IP_3_R inhibitor), muscarine applied without concurrent NMDAR stimulation now potentiated NMDA responses (Fig. 5c,d, xestos: n = 5, *P* < 0.05 compared with baseline). Potentiation of NMDARs by M1R stimulation in the presence of xestospongin C was not influenced by intracellular anti-STEP application, suggesting that block of IP_3_Rs prevents STEP recruitment (Fig. 5c,d, xestos + anti-STEP: n = 5, *P* > 0.05 compared with group without anti-STEP). This suggests that the downstream effect of mAchR stimulation on GluN2ARs is Ca^2+^ source-specific; entry through NMDARs favors Src activation, whilst release from intracellular stores favors STEP activation.

### The activity of STEP and Src is differentially regulated by the concentration and source of intracellular Ca^2+^

Our findings show that the direction of change in GluN2AR function resulting from M1R stimulation is determined by intracellular Ca^2+^ dynamics, presumably by dictating the relative strength of Src vs STEP activity. To confirm the parallel stimulation of Src and STEP activity by M1Rs and consequent change in GluN2A tyrosine phosphorylation, we used Western blotting to examine: (1) Phosphorylation of STEP at Ser221 within the substrate binding domain^28^. Phosphorylation of this site sterically prevents the association of STEP with substrates, resulting in an increase in Tyr phosphorylation of these substrates^29^,^30^,^31^,^32^. (2) Phosphorylation of Src at Tyr416. Residing within the activation loop, phosphorylation at this site activates Src kinase. (3) Phosphorylation of GluN2AR.

**Figure 6:**
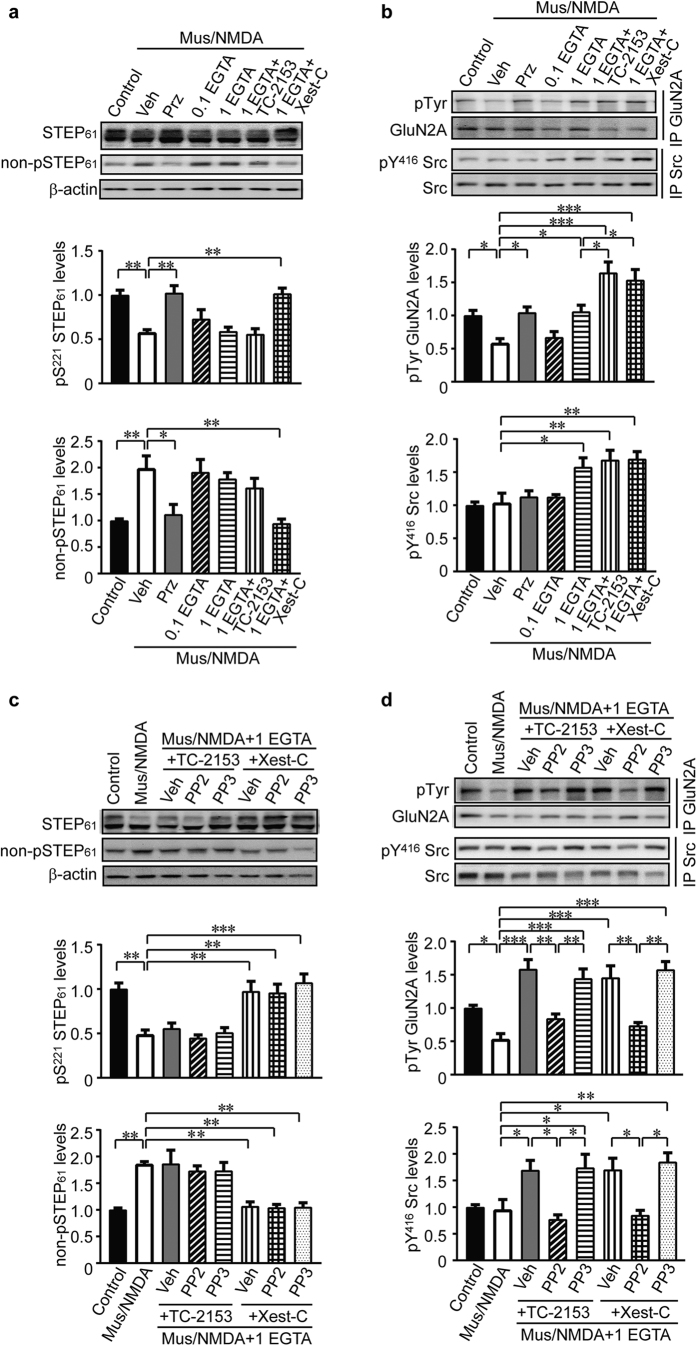
Tyrosine phosphorylation of GluN2A is regulated by STEP_61_ and Src activities. (**a**) Primary hippocampal neurons were pretreated with various inhibitors (veh: 0.1% DMSO; pirenzepine: 10 μM; EGTA/AM: 0.1 or 1 mM; TC-2153: 1 μM; xestospongin C: 10 μM) for 30 min, followed by co-stimulation of muscarine (10 μM) and NMDA (50 μM) for 10 min. Neuronal lysates were probed with anti-STEP (23E5) and anti-non-phospho-STEP_61_ antibodies. Phospho- and non-phospho- STEP_61_ levels were normalized to total STEP_61_ protein levels and then to β-actin as loading control. (**b**) GluN2A and Src were immunoprecipitated from treated lysates using specific antibodies, followed by probing with anti-Tyr, anti-pY416 Src and pan-protein antibodies, respectively. Phospho-protein levels were normalized to pan-protein levels. (**c**) In addition to the inhibitors used in (**a**), PP2 (10 μM) and PP3 (10 μM) were added to ECS for stimulation. Phospho- and non-phospho- STEP_61_ levels were assessed. (**d**) After treatments, phosphorylation of GluN2A and Src were measured on immunoprecipitated samples. Phospho-protein levels were normalized to pan-protein levels. All data were expressed as mean ± SEM. Statistical significance was determined using one-way ANOVA with post hoc Tukey test (*P < 0.05, **P < 0.01, ***P < 0.001, n = 5).

We first treated primary hippocampal neurons with muscarine (10 μM) in the presence of NMDA (50 μM) in ECS for 10 min (Mus/NMDA), without exogenous Ca^2+^ buffers added. Mus/NMDA treatment resulted in decreased phosphorylation of STEP_61_ at Ser221 (Fig. 6a, Control and Veh lanes, n = 5, *P* < 0.01), as revealed by the reduced upper band in the doublets^29^. To confirm this finding we probed using an antibody recognizing STEP_61_ only when not phosphorylated at Ser221. Consistently, we found increased non-phosphorylated STEP_61_ (Fig. 6a, Control and Veh lanes, n = 5, *P* < 0.01), suggesting that Mus/NMDA treatment led to the activation of STEP_61_. In contrast, no change in the phosphorylation of Src at Tyr416 was observed after Mus/NMDA treatment (Fig. 6b, Control and Veh lanes, n = 5, *P* > 0.05). To investigate changes in GluN2A upon Mus/NMDA treatment, we immunoprecipitated GluN2A followed by probing with anti-pan-Tyr antibody. We found decreased Tyr phosphorylation of GluN2A (Fig. 6b, Control and Veh lanes, n = 5, *P* < 0.05), in agreement with the inhibition of NMDAR response. The effects of muscarine were prevented when applied in the presence of the M1R antagonist pirenzepine (Fig. 6a,b, Prz lanes, n = 5, *P* < 0.05, *P* < 0.01, respectively).

Next, we examined whether STEP_61_, Src and GluN2A phosphorylation was altered by pre-treatment with cell-permeable EGTA/AM prior to Mus/NMDA application. The increase in STEP_61_ activity (i.e. reduced Ser221 phosphorylation) induced by Mus/NMDA was not affected by either concentration of EGTA/AM tested (0.1 and 1 mM; Fig. 6a, 0.1 EGTA and 1 EGTA lanes, n = 5, *P* > 0.05 compared with Mus/NMDA alone). However, the higher (1 mM) but not the lower (0.1 mM) concentration of EGTA/AM reversed Mus/NMDA-induced decreases in pTyr GluN2A, possibly via the activation of Src (Fig. 6b, 1 EGTA, n = 5, *P* < 0.05). These results are consistent with our electrophysiological findings whereby 1 mM but not 0.1 mM EGTA could prevent inhibition of NMDA currents by muscarine (Fig. 4a,b,d). Recordings with 1 mM EGTA (Fig. 4a,d) revealed that Mus/NMDA treatment enhanced NMDAR currents when STEP is inhibited. To test whether inhibition of STEP_61_ would enhance Tyr phosphorylation of GluN2A we used a recently characterized STEP inhibitor TC-2153^33^. STEP inhibition by TC-2153 enhanced Tyr phosphorylation of GluN2A (Fig. 6b, 1 EGTA + TC-2153, n = 5, *P* < 0.05 compared with 1 mM EGTA) without altering phosphorylation of Src (Fig. 6b, 1 EGTA + TC-2153, n = 5, *P* > 0.05 compared with 1 EGTA). A comparable increase in GluN2A Tyr phosphorylation was observed when Mus/NMDA was applied in the presence of the IP_3_R blocker, xestospongin C (Fig. 6b, 1 EGTA + Xest-C, n = 5, *P* < 0.05 compared with 1 mM EGTA). Xestospongin C, but not TC-2153, also normalized levels of phosphorylated and non-phosphorylated form of STEP_61_ (Fig. 6a, 1 EGTA + Xest-C and 1 EGTA + TC-2153, n = 5, *P* < 0.01, *P* > 0.05, respectively). Src phosphorylation was not affected by xestospongin C.

**Figure 7:**
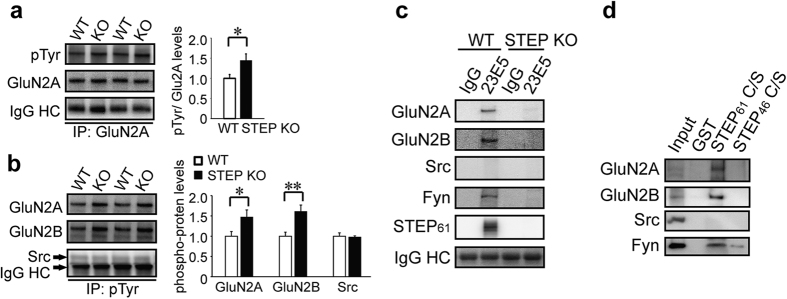
STEP binds to and dephosphorylates GluN2A. Synaptosomal fractions from WT and STEP KO mouse hippocampus were used for immunoprecipitation with anti-GluN2A (**a**) or anti-phophos-Tyr (**b**), followed by immunoblotting with anti-phopho-Tyr or anti-GluN2A, anti-GluN2B and anti-Src, respectively. Phospho-protein levels were normalized to total proteins, repectively. All data were expressed as mean ± SEM. Statistical significance was determined using two-tailed Student’s t test (*P < 0.05, **P < 0.01, n = 6). (**c**) STEP was immunoprecipitated from WT and STEP KO hippocampal lysates using anti-STEP (23E5) antibody. Potential interacting proteins were verified using selective antibodies indicated in the figure. Representative blots were shown from three independent replicates. (**d**) GST-STEP fusion proteins were bound to glutathione-sepharose 4B beads and incubated with hippocampal lysates. Co-purified proteins were verified using selective antibodies indicated in the figure. Representative blots were shown from three independent replicates.

To demonstrate the role of Src in mediating the increase in GluN2A Tyr phosphorylation when STEP is inhibited by TC-2153 or xestospongin C in neurons pre-treated with 1 mM EGTA/AM, we repeated Mus/NMDA co-stimulation in the presence of Src inhibitor PP2 or the negative control PP3. Inhibition of Src by PP2 completely blocked the increase in the Tyr phosphorylation of GluN2A after STEP inhibition by xestospongin C and TC-2153, while PP3 had no effect (Fig. 6d, top panel, PP2: PP2 vs. Veh (+TC-2153) and PP2 vs Veh (+Xest-C), n = 5, *P* < 0.01 respectively; PP3: PP3 vs. Veh (+TC-2153) and PP3 vs. Veh (+Xest-C), n = 5, *P* > 0.05, respectively). PP2 but not PP3 also blocked phosphorylation of Src at Tyr416 (Fig. 6d, bottom panel, PP2: PP2 vs. Veh (+TC-2153) and PP2 vs Veh (+Xest-C), n = 5, *P* < 0.05, respectively; PP3: PP3 vs. Veh (+TC-2153) and PP3 vs. Veh (+Xest-C), n = 5, *P* > 0.05, respectively), whilst STEP phosphorylation was unaffected by PP2 or PP3 (Fig. 6c). To provide additional evidence that STEP regulates GluN2A tyrosine phosphorylation, we used a pan-Tyr antibody on GluN2A immunoprecipitates from WT and STEP KO hippocampus. We observed increased Tyr phosphorylation of GluN2A but not of total GluN2A protein levels in the synaptosomal fraction (Fig. 7a, n = 6, *P* < 0.05). We also performed immunoprecipitation with anti-Tyr antibody and found increased presence of GluN2A in STEP KO hippocampal lysates (Fig. 7b, n = 6, *P* < 0.05), indicating an increase in its Tyr phosphorylation. The presence of GluN2B, but not of Src, was similarly increased in these samples (Fig. 7b, n = 6, *P* < 0.01, *P* > 0.05, respectively).

Previous studies have shown that Fyn, but not Src, is a STEP substrate^32^,^34^. Evidence also suggests an interaction between STEP_61_ and GluN2A in heterologous cell lines^35^. Thus we set out to test whether there is a direct link between STEP_61_ and GluN2A. We immunoprecipitated STEP using a well-validated anti-STEP (23E5) antibody and looked for co-immunoprecipitation of interacting proteins. We confirmed previous findings that GluN2A, but not Src, is associated with STEP_61_ in hippocampal lysates (Fig. 7c, first 2 lanes, n = 3). As expected, known substrates GluN2B and Fyn also bind to STEP_61_ (Fig. 7c, first 2 lanes, n = 3). We also used STEP KO hippocampal lysates to exclude possible non-specific binding (Fig. 7c, last 2 lanes, n = 3). To confirm the interaction between STEP_61_ and GluN2A we used an *in vitro* binding assay^31^,^34^. A substrate-trapping mutant of the longer isoform (STEP_61_) and the shorter isoform (STEP_46_) were immobilized on sepharose matrix, and incubated with mouse hippocampal lysates. We found co-purification of GluN2A with STEP_61_ but not STEP_46_, suggesting the extra N-terminus in STEP_61_ is required for the binding (Fig. 7d). In agreement with previous findings^31^,^34^ GluN2B and Fyn also showed higher affinity with STEP_61_ (Fig. 7d). We didn’t find interaction of Src and STEP_61_ under these conditions (Fig. 7d). These findings confirm that not only GluN2B and Fyn but also GluN2A are substrates of STEP.

## Discussion

**Figure 8:**
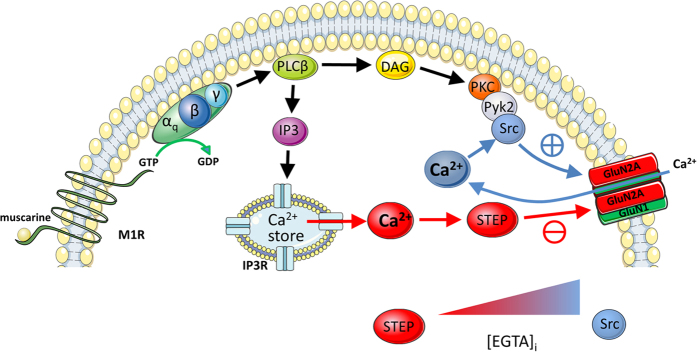
A summary diagram depicting the concurrent activation of both Src and STEP downstream of M1 muscarinic acetycholine receptor (M1R) stimulation. Muscarine stimulation of M1Rs can potentiate GluN2AR containing NMDARs (GluN2ARs) via a sequential cascade leading to the recruitment of Src and increased tyrosine phosphorylation of GluN2A subunits. Potentiation of GluN2AR by Src is facilitated by Ca^2+^ entry via NMDARs. Muscarine can depress GluN2ARs via IP_3_R-dependent recruitment of STEP leading to decreased tyrosine phosphorylation of GluN2A subunits. The balance between Src and STEP activation can be altered by varying the intracellular concentration of EGTA ([EGTA]_i_); increased [EGTA]_I_ favors Src- over STEP-mediated regulation of GluN2ARs.

Here we show that stimulation of Gαq-coupled receptors for muscarine (M1R) or pituitary adenylate cyclase activating peptide (PAC_1_R) potentiate NMDAR-mediated currents. The enhancement of NMDA currents was mediated by Src acting specifically upon NMDARs composed of the GluN2A receptor subunit. A major finding of our study is that the Src-mediated increase in GluN2AR function is counterbalanced by STEP activated concurrently by Gαq receptors. Under conditions that favor Src activation, STEP limits the potentiation of GluN2ARs by Src. Conversely, under conditions that favor STEP activation, STEP depresses the function of GluN2ARs (Fig. 8). Importantly, we show that potentiation of NMDAR currents by Src and inhibition by STEP downstream of M1Rs have discreet Ca^2+^ requirements; Src requires entry of Ca^2+^ via NMDARs whereas STEP requires release of Ca^2+^ from IP_3_R-sensitive stores. The balance of Src and STEP activation, and consequent impact on GluN2AR function, is dictated by the dynamic balance between source specific intracellular Ca^2+^ elevations. More conclusively, we monitored changes in the phosphorylation of STEP and Src at key sites that regulate their enzyme activity and show that co-stimulation of M1R and NMDARs can cause both STEP and Src activation. Consistent with electrophysiological findings, the direction of change in GluN2A tyrosine phosphorylation was determine by the relative strength of Src or STEP activation; conditions that caused increased NMDAR current with M1R stimulation favored Src activation and increased GluN2A tyrosine phosphorylation and, conversely, conditions that decreased NMDAR currents with M1R stimulation favored STEP activation and decreased GluN2A tyrosine phosphorylation.

In contrast, when Gαs-coupled D1Rs were stimulated the resulting Fyn-dependent enhancement of GluN2BRs was not influenced by STEP. We attribute this to several factors. Firstly, past work has shown that Gαs-coupled D1Rs inhibit STEP via PKA-mediated phosphorylation at Ser221^28^,^36^ within the kinase-interacting motif (KIM) domain important for STEP substrate recognition. Secondly, our own results demonstrate that basal NMDAR function is not influenced by STEP, consistent with evidence that STEP activity is low under resting conditions^29^,^37^. Thus, the parallel recruitment of Fyn, in concert with suppression of low basal STEP activity, accounts for the observed D1R-mediated enhancement of GluN2BR function that is unopposed by STEP (Supplementary Fig. 3). Accordingly, key to reconciling divergent NMDAR subunit- and SFK-selective actions of STEP is to consider the activation context. For example, β-amyloid provokes increased STEP levels in Alzheimer’s disease through inhibition of the proteasome that normally degrades STEP, resulting in GluN2B internalization as a consequence of STEP-mediated dephosphorylation of Tyr1472^18^,^19^,^20^. Our findings suggest an additional and previously overlooked context in which STEP is recruited by Gαq receptors (e.g. PAC_1_R and M1R). Unlike the D1R pathway in which Fyn signalling is augmented through inhibition of STEP, pathways downstream of PAC_1_R and M1R initiate an increase in the activity of both Src and STEP. In this way STEP provides feedback inhibition that constrains enhancement of NMDAR function through concurrent Src activation targeting GluN2ARs.

The concurrent stimulation of Src and STEP by Gαq receptors allows for bidirectional modulation of NMDAR function. We find that intracellular Ca^2+^ dynamics and the source contributing to intracellular Ca^2+^ elevations determines the direction of change in GluN2AR function. This was evident from M1R stimulation experiments in which we varied Ca^2+^ buffering by EGTA allowing the extent of intracellular Ca^2+^ elevations to be experimentally determined. Whereas robust Src-dependent potentiation is observed when large elevations of intracellular Ca^2+^ are prevented (11 mM EGTA), M1R stimulation fails to potentiate the NMDA responses when modest intracellular Ca^2+^ concentrations are achieved (1 mM EGTA; Fig. 4a,d). Nevertheless, when modest intracellular Ca^2+^ elevations are permitted, treatment with anti-STEP enables muscarine to now potentiate the NMDA response, whereas treatment with Src(40–58) enables muscarine to now depress the NMDA response. When large intracellular Ca^2+^ elevations are permitted (0.1 mM EGTA; Fig. 4b,d), muscarine now depresses NMDA responses and this depression can be prevented by treatment with anti-STEP. In considering the functional outcome for GluN2ARs, these results indicate that the balance between Src-mediated potentiation and STEP-mediated depression is determined by intracellular Ca^2+^ levels. This model is precisely supported by our biochemical findings in which we varied intracellular Ca^2+^ buffering using cell-permeable EGTA/AM. Mus/NMDA treatment depresses GluN2A tyrosine phosphorylation in neurons pre-treated with 0.1 mM EGTA/AM, but not in neurons pre-treated with 1 mM EGTA. Of note, the difference between these two conditions resides in the sensitivity of Src activity to the concentration of applied EGTA/AM. In the presence of 1 mM EGTA/AM, Mus/NMDA increased Src Tyr416 phosphorylation whereas no change in Src Tyr416 was observed when Mus/NMDA was applied in the presence of 0.1 mM EGTA/AM. As increasing the concentration of EGTA/AM from 0.1 to 1 mM is anticipated to reduce the rise in intracellular Ca^2+^ achieved during Mus/NMDA treatment, these findings suggest that a Ca^2+^ -dependent tyrosine phosphatase may limit Src activity. Although the phosphatase identity remains to be determined, collectively our biochemical findings rule out a role for STEP given convincing evidence that Src is not a STEP substrate.

A noteworthy aspect of our findings is the observed requirement of coincident mAchR and GluN2AR stimulation for Src-mediated potentiation of GluN2ARs. A similar requirement was previously reported for the mGluR5-induced enhancement of NMDAR function^38^. This was demonstrated through experiments in which muscarine was applied in the absence of co-incident NMDAR stimulation. Here, muscarine did not potentiate NMDA responses but could do so in the presence of anti-STEP. This suggests that STEP was dominant under these conditions. In considering the Ca^2+^ source contributing to STEP recruitment in the absence of NMDAR stimulation, a likely source was through mobilization of internal Ca^2+^ stores by IP_3_Rs. This was confirmed by demonstrating that muscarine, applied without concurrent NMDAR stimulation, could potentiate NMDAR currents in the presence of the IP_3_R blocker xestospongin C. Confirming that STEP was inactive, anti-STEP treatment was ineffective when applied with IP_3_Rs blocked. Our findings suggest a previously overlooked aspect of STEP function, namely the feedback regulation of GluN2AR function augmented by Src signalling downstream of Gαq-coupled GPCRs. Although we show that STEP activation reduces GluN2A tyrosine phosphorylation, the mechanism by which this biochemical change inhibits GluN2AR function remains to be determined. Additional experiments (Supplementary Fig. 4) suggest that it does not involve changes in tyrosine phosphorylation of GluN2A Tyr1325. Speculatively, a mechanism can be proposed on the basis of past work demonstrating that dephosphorylation of GluN2A Tyr842 depresses NMDA currents through AP2 mediated receptor internalization^39^. Such a mechanism would be analogous to that by which STEP supresses the function of GluN2BRs through dephosphorylation of GluN2B at Tyr1472 and receptor internalization^18^,^20^.

The present findings and past work, which has demonstrated the important role of STEP in offsetting Fyn-mediated phosphorylation of GluN2B subunits, provides a broader context in which STEP can be recruited to limit NMDAR function augmented under different physiological and pathophysiological conditions by SFK signalling. Our study used fast NMDA applications to acutely isolated hippocampal neurons as a means of assessing the consequence of Src and STEP recruitment on NMDAR function. The NMDA responses were therefore likely mediated primarily by extrasynaptic NMDARs. Nevertheless, all of the signalling components examined regulate synaptic NMDARs^17^. Therefore, contingent on the basal phosphorylation status of NMDAR subunits, Src and/or STEP, our findings suggest that stimulation of mAchR (or other Gαq-coupled GPCRs) at quiescent glutamate synapses will favor STEP recruitment leading to reduced function of GluN2ARs. Conversely, Src activation will be favored at active glutamate synapses leading to augmented GluN2AR function. Additionally, spontaneous synaptic events promoting Ca^2+^ entry via NMDARs may participate in maintaining basal Src activity necessary for homeostatic maintenance of synaptic NMDAR functions. Considered in light of past evidence demonstrating that D1Rs augment GluN2BR function via Fyn stimulation and STEP inhibition, these findings suggest that STEP, acting in concert with SFKs, dynamically orchestrates NMDAR subunit-dependent signalling downstream of GPCRs. The functional interplay between SFKs and STEP can be finely regulated at individual synapses based on the level of activity and whether such activity converges with input from transmitter systems acting upon their cognate GPCRs. This functional interplay is likely to have important implications for regulating the direction of plasticity and thus metaplasticity at excitatory synapses.

## Methods

### Cell isolation and whole-cell recordings

Hippocampal CA1 neurons were isolated from Wistar rats (14–22 days old) or from STEP KO mice and WT littermates (21–28 days old) using previously described procedures^6^,^40^. All animal experimentations were conducted in accordance with standards established by the Canadian Council on Animal Care (CCAC) and approved by the Animal Care Committee at the University of Western Ontario. The extracellular solution (pH 7.4, osmolality between 315 and 325 mOsm) consisted of the following (in mM): 140 NaCl, 1.3 CaCl_2_, 5 KCl, 25 HEPES, 33 glucose, 0.0005 glycine and 0.0005 tetrodotoxin. Recording electrodes (resistance between 3 and 5 MΩ) were constructed from borosilicate glass (1.5 mm in diameter, World Precision Instruments, Sarasota, FL) using a two-stage puller (PP83, Narishige, Tokyo, Japan) and were filled with intracellular solution (pH 7.2, osmolality between 290 and 300 mOsm) containing the following (in mM): 140 CsF, 1 CaCl_2_, 2 MgCl_2_, 10 HEPES, 2 tetraethylammonium, and 2 K_2_ATP. Unless otherwise indicated, the EGTA concentration used in intracellular solution is 11 mM EGTA. Where indicated, 1 mM or 0.1 mM EGTA was used instead. For some experiments, the intracellular solution was supplemented with Src(40–58), Fyn(39–57), anti-STEP, orthovanadate or xestospongin C, with all concentrations indicated in the text. Recordings were conducted at room temperature between 20–22 °C. After formation of the whole-cell configuration, the neurons were voltage clamped at −60 mV and lifted into the stream of solution supplied by a computer-controlled multi-barreled fast perfusion system (SF-77 B, Warner Instrument Corporation). The solution exchange time was 3–5 ms. NMDA (50 μM) was applied 1/60 sec for 3 sec. To monitor access resistance, a voltage step of −10 mV was applied before each application of NMDA (50 μM). When series resistance increased to >20 MΩ, the cell was discarded. Currents were recorded using an Axopatch 1D amplifier. Data were filtered at 2 kHz and digitized at 10 kHz using Clampex software.

### Transgenic mice and sample preparation

The STEP knockout (KO) mice and wild-type (WT) littermates were generated and maintained at Yale University as described previously^41^. All procedures were performed in accordance to the National Institutes of Health Guide for the Care and Use of Laboratory Animals and were approved by the Institutional Animal Care and Use Committee at Yale University. Mouse (both male and female, 3–6 months old) hippocampi were dissected out and homogenized in Dounce tissue grinders (Wheaton, Millville, NJ) in ice-cold TEVP buffer (10 mM Tris pH 7.4, 1 mM EDTA, 1 mM EGTA, 1 mM Na_3_VO_4_, 5 mM NaF, 320 mM sucrose) containing complete protease and phosphatases inhibitor cocktails (Roche, Nutley, NJ) as described previously^33^. Homogenates were spun at 12,000 × g for 15 min to obtain synaptosomal fractions.

### Primary hippocampal cultures and treatments

All procedures were approved by the Yale University Institutional Animal Care and Use Committee. Primary dissociated hippocampal cultures were prepared from Sprague–Dawley rat at embryonic day 18 and plated on poly-D-lysine-coated plates (1 × 10^6^ cells/well) in Neurobasal supplemented with 2% B27 (Invitrogen, San Diego, CA) and grown for 18–21 days as described^33^. At 18–21 days *in vitro*, neurons were treated with muscarine (10 μM) plus NMDA (50 μM) dissolved in ECS for 10 min. In some groups, inhibitors and antagonists (pirenzepine: 10 μM; EGTA/AM: 0.1 or 1 mM; TC-2153: 1 μM; xestospongin C: 10 μM; PP2: 10 μM or PP3: 10 μM) or vehicle (0.1% DMSO) were added 30 min before and throughout the treatment with muscarine and NMDA. After treatments, neurons were lysed in 1 × RIPA buffer (Pierce Biotechnology, Rockford, IL) with complete phosphatase and protease inhibitors (Roche, Indianapolis, IN).

### Immunoprecipitation

Lysates were spun at 1,000 × g for 10 min to remove insoluble debris. Protein content in supernatant was determined using BCA protein assay kit (Pierce). Three hundred μg of lysates were first precleared with protein A/G-agarose beads (Santa Cruz Biotechnology, Santa Cruz, CA) and mixed with anti-GluN2A or anti-Src antibodies (Millipore, Bedford, MA) overnight at 4 °C. On the second day, protein A/G-agarose was added to the antibody-bound complex for 4 h at 4 °C. The precipitates were washed 3 times with 1 × RIPA buffer and resuspended in heated 2× Laemmli sample buffer (Bio-Rad, Richmond, CA). In some experiments, synaptosomal fractions from WT and STEP KO mouse hippocampus were resuspended in 1× RIPA buffer and followed the immunoprecipitation procedures with anti-GluN2A or anti-phospho-Tyr antibodies (Millipore).

### Co-immunoprecipitation and GST pull-down

Synaptosomal fractions from WT and STEP KO mouse hippocampus were lysed in co-IP buffer (20 mM Tris–HCl, pH 8, 150 m NaCl, 0.5% NP-40, proteases and phosphatase inhibitors) and subjected to immunoprecipitation with anti-STEP antibody (Millipore) overnight at 4 °C. The second day protein A/G plus agarose beads (Santa Cruz) were added and incubated for another 4 h. Beads were collected by centrifugation and washed 3 times with co-IP buffer. Immunoprecipitates were resuspended in heated 2× Laemmli sample buffer (Bio-Rad).

For GST pull-down assays, GST-STEP fusion constructs were cloned and expressed in *E. coli* BL21 (DE3) and conjugated to glutathione-sepharose 4B beads (GE Lifesciences, Piscataway, NJ) as described previously^31^,^34^. Proteins (1 μg) were incubated with hippocampal synaptosomal lysates (100 μg) in co-IP buffer overnight at 4 °C. Beads were washed 3 times with co-IP buffer and resuspended in heated 2× Laemmli sample buffer.

### Western blotting

Samples were resolved on 8% SDS-PAGE, transferred onto nitrocellulose membranes (Bio-Rad), and incubated with primary antibodies against STEP (1:1000, Millipore), non-phospho-STEP (1:1000, Cell Signaling), phospho-Tyr (1:2000, Millipore), GluN2A (1:2000, Millipore), GluN2B (1:2000, Millipore), pY416 Src (1:1000, Cell Signaling), Src (1:2000 Santa Cruz), Fyn (1:2000 Santa Cruz), and β-actin (1:5000, Santa Cruz) overnight at 4 °C, and HRP-conjugated secondary antibodies (1:5000; Pierce) or Clean-Blot IP detection reagent HRP (1:1000; Thermo Scientific, Rockford, IL) for 2 h at RT. Immunoreactivity was developed with a Chemiluminescent Substrate kit (Pierce) and visualized by G:BOX with the GeneSnap software (Syngene, Cambridge, UK). All densitometric quantification was obtained using ImageJ software (National Institutes of Health).

### Data analyses

For electrophysiological experiments, all population data are expressed as mean ± SEM. Normalized values represent the average of NMDA receptor-mediated peak current amplitudes recorded between 20–25 min normalized to the average of peak current amplitudes recorded during the first 5 min of recording, prior to Gαq-coupled GPCR agonist application. For statistical analysis of recordings from neurons that served as their own control (i.e. responses recorded before and after drug treatment), paired Student’s t-test was used. Unpaired Student’s t-test was used to compare between two groups and one-way ANOVA (Tukey’s post hoc comparison) was used to compare multiple groups. For biochemistry, all experiments were repeated at least three times. Data were expressed as means ± SEM. Statistical significance (*P* < 0.05) for biochemical data was determined by one-way ANOVA with post hoc Tukey test and two-tailed Student’s t-test.

### Drugs and peptides

Muscarine, Ro25-6981, NMDA, glycine, carbachol, PACAP38, sodium orthovanadate, SKF81297 and DMSO were bought from Sigma (St Louis, MO). TCN201, pirenzepine and xanomeline oxalate were bought from Tocris Bioscience (Bristol, UK). Xestospongin C, PP2 and PP3 were bought from Cayman Chemicals (Ann Arbor, MI). Src(40–58) were provided by Dr. MW Salter (Hospital for Sick Children, Toronto, Canada). Fyn(39–57) was synthesized by the Advanced Protein Technology Centre (Toronto, Canada). PACAP and cell permeable EGTA/AM were from Calbiochem (San Diego, CA). The STEP inhibitor TC-2153 was synthesized as described previously^33^.

## Additional Information

**How to cite this article**: Tian, M. *et al*. STEP activation by Gαq coupled GPCRs opposes Src regulation of NMDA receptors containing the GluN2A subunit. *Sci. Rep.* **6**, 36684; doi: 10.1038/srep36684 (2016).

**Publisher’s note:** Springer Nature remains neutral with regard to jurisdictional claims in published maps and institutional affiliations.

## Supplementary Material

Supplementary Information
