# Supplementary material for: STEP activation by Gαq coupled GPCRs opposes Src regulation of NMDA receptors containing the GluN2A subunit
Source: Sci Rep. 2016 Nov 18;6:36684. doi: 10.1038/srep36684 (PMC5114553; doi:10.1038/srep36684)
Supplement: Supplementary Information [file srep36684-s1.pdf]

STEP activation by Gαq coupled GPCRs opposes Src regulation of NMDA receptors containing the GluN2A subunit

Meng Tian[1], Jian Xu[5], Gang Lei[1], Paul J. Lombroso[5,6,7], Michael F. Jackson[3,4,*] and John F. MacDonald[1,2, †]

[1]Molecular Medicine, Robarts Research Institute, [2]Department of Physiology and Pharmacology, Schulich School of Medicine, the University of Western Ontario, London, Ontario, N6A 5B7, [3]Department of Pharmacology and Therapeutics, College of Medicine, University of Manitoba, Winnipeg, Manitoba R3E 0T6, [4]Neuroscience Research Group, Kleysen Institute for Advanced Medicine, University of Manitoba, Winnipeg, Manitoba, R3E 3J7, , [5]Child Study Center and Departments of [6]Psychiatry, and [7]Neuroscience, Yale University School of Medicine, 230 South Frontage Rd, New Haven, CT, 06520.

† Deceased, April 22nd, 2014

* Corresponding author: michael.jackson@umanitoba.ca

Phone: 204-789-3951

Supplementary Figure S1

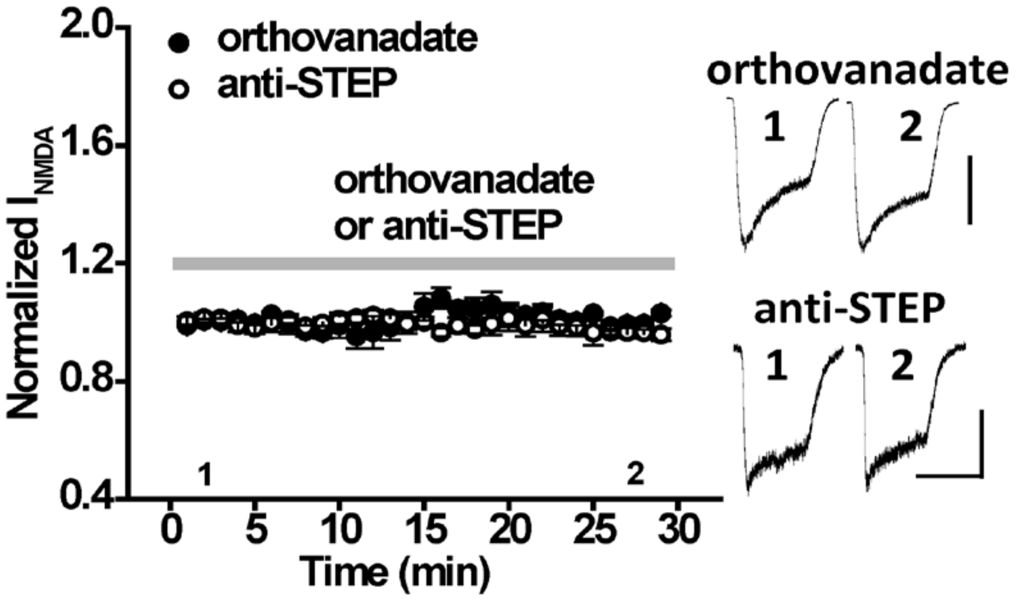

Supplementary Figure S1. **Tyrosine phosphatase or STEP inhibition alone does not influence NMDAR currents.** When applied alone, neither the non-specific tyrosine phosphatase inhibitor orthovanadate (10 µM; n = 4, 1.00 ± 0.01, closed circle, *P* > 0.05 compared with baseline) nor the STEP specific antibody anti-STEP (1:400 dilution; n = 4, 0.96 ± 0.02, *P* > 0.05 compared with baseline) has any effect on NMDA currents. Calibration bars: 3s; orthovanadate 400 pA, anti-STEP 200 pA.

Supplementary Figure S2

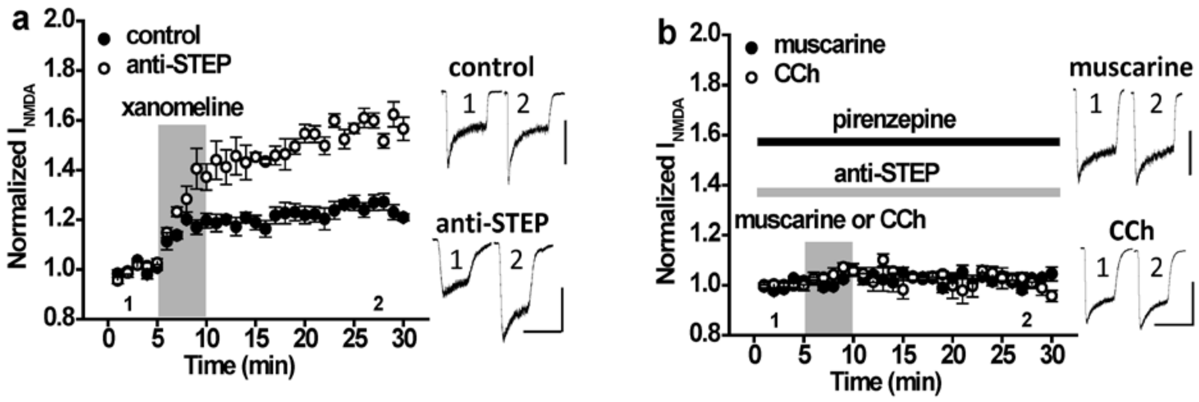

Supplementary Figure S2. **NMDARs are potentiated via M1 mAChR stimulation.** (**a**) Xanomeline application (10 µM; timing indicated by the shaded region) potentiates NMDAR currents (control: n = 5, 1.24 ± 0.02). Xanomeline potentiation of NMDAR current is further increased by anti-STEP application (anti-STEP: n = 5, 1.59 ± 0.03, $P < 0.05$ compared with xanomeline). (**b**) In the presence of pirenzepine (10 µM), muscarine (10 µM; n =3, 1.00 ± 0.05, $P > 0.05$ compared with baseline) or CCh (5 µM; n =4, 1.01 ± 0.01, $P > 0.05$ compared with baseline) application had no effect on NMDA currents despite inclusion of anti-STEP. Calibration bars: 3s; (**a**) control 250 pA, anti-STEP 400 pA; (**b**) muscarine 200 pA, CCh 500 pA.

Supplementary Figure S3

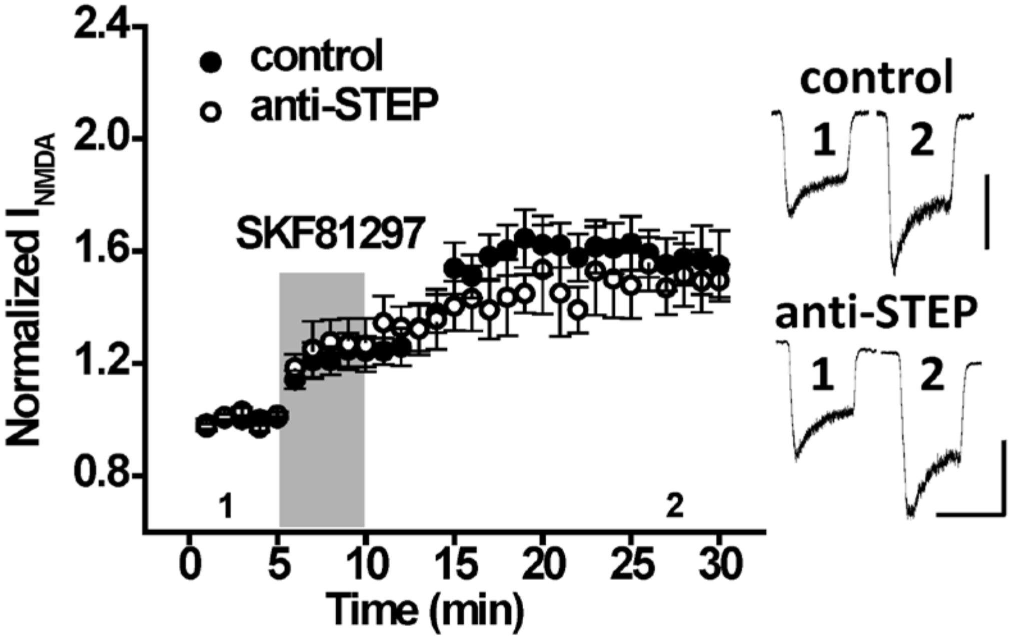

Supplementary Figure S3. **STEP inhibition does not affect potentiation of NMDA currents by D1R stimulation.** SKF81297 application (10 µM; timing indicated by the shaded region) potentiates NMDAR current (n = 6, 1.56 ± 0.11, $P < 0.05$ compared with baseline). Intracellularly applied anti-STEP has no effect on SKF81297 potentiation of NMDAR currents (n = 6, 1.50 ± 0.10, $P > 0.05$ compared with SKF81297). Calibration bars: 3s; control 350 pA; anti-STEP 250 pA.

Supplementary Figure S4

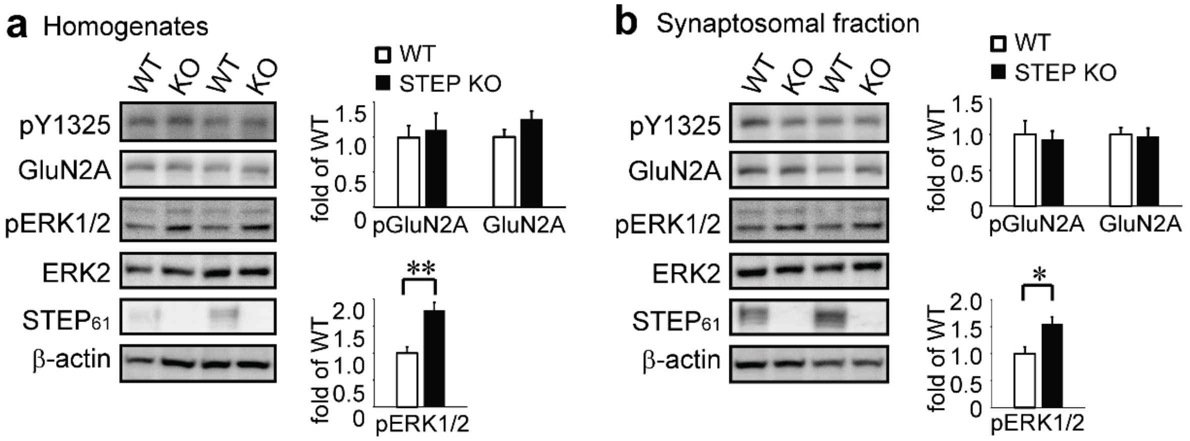

Supplementary Figure S4. Phosphorylation of GluN2A at Tyr1325 is not altered in STEP KO mouse hippocampus. Wild type (WT) and STEP knockout (KO) mouse (3-6 months old) hippocampi were dissected out and processed to obtain homogenates (**a**) and synaptosomal fractions (**b**). Blots were probed with anti-pY1325 GluN2A, anti-pY204/187 ERK1/2 and pan protein antibodies, respectively. Phospho-protein levels were normalized to total proteins, and then to β-actin as loading control. All data were expressed as mean ± SEM. Statistical significance was determined using two-tailed Student's t test (**P* < 0.05, ***P* < 0.01, n = 6).
